# Thermal Fatigue Behavior of Air-Plasma Sprayed Thermal Barrier Coating with Bond Coat Species in Cyclic Thermal Exposure

**DOI:** 10.3390/ma6083387

**Published:** 2013-08-08

**Authors:** Zhe Lu, Sang-Won Myoung, Yeon-Gil Jung, Govindasamy Balakrishnan, Jeongseung Lee, Ungyu Paik

**Affiliations:** 1 School of Nano & Advanced Materials Engineering, Changwon National University, Changwon, Gyeongnam 641-773, Korea; E-Mails: lz19870522@126.com (Z.L.); msw1980@changwon.ac.kr (S.-W.M.); 2 School of Mechatronics, Changwon National University, #9 Sarim-dong, Changwon, Gyeongnam 641-773, Korea; E-Mail: balaphysics76@gmail.com; 3 Department of Mechnical Engineering, Bharath University, Chennai, Tamilnadu-600073, India; 4 Materials Engineering Group, Energy Equipment R&D Center, Samsumg Techwin Co. Ltd., Seongnam, Gyeonggi 463-400, Korea; E-Mail: jsdaniel.lee@samsung.com; 5 Department of Energy Engineering, Hanyang University, Haengdang-dong, Sungdon-gu, Seoul 133-791, Korea

**Keywords:** thermal barrier coating, bond coat, air-plasma spray, thermal durability, cyclic thermal exposure, thermal-shock

## Abstract

The effects of the bond coat species on the delamination or fracture behavior in thermal barrier coatings (TBCs) was investigated using the yclic thermal fatigue and thermal-shock tests. The interface microstructures of each TBC showed a good condition without cracking or delamination after flame thermal fatigue (FTF) for 1429 cycles. The TBC with the bond coat prepared by the air-plasma spray (APS) method showed a good condition at the interface between the top and bond coats after cyclic furnace thermal fatigue (CFTF) for 1429 cycles, whereas the TBCs with the bond coats prepared by the high-velocity oxygen fuel (HVOF) and low-pressure plasma spray (LPPS) methods showed a partial cracking (and/or delamination) and a delamination after 780 cycles, respectively. The TBCs with the bond coats prepared by the APS, HVOF and LPPS methods were fully delaminated (>50%) after 159, 36, and 46 cycles, respectively, during the thermal-shock tests. The TGO thickness in the TBCs was strongly dependent on the both exposure time and temperature difference tested. The hardness values were found to be increased only after the CFTF, and the TBC with the bond coat prepared by the APS showed the highest adhesive strength before and after the FTF.

## 1. Introduction

Thermal barrier coatings (TBCs) have been applied to the hot components of engines because of the increasing demands for higher gas turbine engine performance. The TBCs can be considered as a three-layered material system, consisting of (1) a substrate (nickel- or cobalt-based superalloy); (2) an oxidation-resistant metallic bond coat (MCrAlY or a platinum aluminide coating); and (3) a ceramic top coating (6–8 wt % yttria-stabilized zirconia) deposited either by the air-plasma spray (APS) or electron beam–physical vapor deposition (EB–PVD) process. A thermal-spraying process, such as APS, twin wire-arc spraying, and high-velocity oxygen fuel (HVOF) spraying, is the most popular deposition technology from an economic point of view and involves many small particles being accelerated by the high-power plasma or combustion flow to form a coating layer. It is well known that many new techniques, such as solution-precursor plasma spraying and electron beam–directed vapor deposition, have exhibited increasing potential in improving the thermal durability of thermal barrier coating (TBC) systems [1,2,3,4].

The common processes used to deposit the ceramic top coat are APS and EB–PVD. The EB–PVD coating has been developed to obtain a good microstructure, enhance adhesive strength, and improve strain resistance. The APS coating with its economic benefits is still preferred commercially, in contrast to the use of the complex and expensive EB–PVD [5,6,7], although it has a low strain tolerance compared with coatings created by more advanced coating methods. The bond coat plays an important role in ensuring structural effectiveness and affording extra adhesion of the top coat to the substrate. Many techniques have been applied to form the bond coat, such as low-pressure plasma spray (LPPS), APS, high-frequency pulse detonation, and HVOF spray [8,9,10,11,12]. The APS process is widely used to create the bond coat in a TBC system because of its economic benefits. However, to meet the requirement for increased working temperature and for improved fuel efficiency in gas turbines and diesel engines, the HVOF process is employed for the bond coat. Unfortunately, the high temperatures and oxidation environment required for the HVOF spray process may affect the subsequent oxidation properties of the top coat, which are important in the applied high-temperature working environment. A dense bond coat without oxide formation during spraying can be deposited by LPPS [13]. Therefore, the bond coat prepared by LPPS has been employed in the most advanced TBCs [14,15,16,17], although their wide application is limited because of their high costs.

The performance of each coating in the bond and top coats has been studied extensively for several decades and a new technology or an advanced TBC has been proposed. However, the best combination of the bond and top coats to improve the thermomechanical properties and to enhance the thermal durability simultaneously is not yet available. Therefore, in the present study, the effects of bond coat species on the thermal durability of TBC systems were investigated through three kinds of cyclic thermal exposure, including delamination or fracture behavior of the TBC systems. Three types of bond coat were prepared using the three different processes of APS, HVOF, and LPPS. The microstructure evolution, mechanical properties, and fracture behavior of all of the TBC systems were compared before and after cyclic thermal exposure.

## 2. Experimental Procedure

### 2.1. Preparation of TBC Specimens

The nickel-based superalloy GTD-111 was used as a substrate. The GTD-111 superalloy has the following nominal composition by weight: Ni = 60.36%, Cr = 14.0%, Co = 9.5%, Ti = 4.9%, W = 3.8%, Al = 3.0%, Ta = 2.8%, Mo = 1.5%, C = 0.1%, Zr = 0.03%, and B = 0.01%. The dimensions of the substrate were 25.4 mm diameter and 5 mm thickness. The substrate was sandblasted using an Al_2_O_3_ powder, and then the coating processes for the bond and top coats were conducted within 2 h. Two types of feedstock powder with different particle sizes and distributions were used to coat the bond coat onto the substrate: AMDRY 962 (Sulzer Metco Holding AG, Switzerland, nominal composition of Ni–22Cr–10Al–1.0Y in wt % and particle size of 56–106 μm) for the APS process and AMDRY 9951 (Sulzer Metco Holding AG, nominal composition of Co–32Ni–21Cr–8Al–0.5Y in wt % and particle size of 5–37 μm) for the HVOF and LPPS processes. The thickness of the bond coat was approximately *d* = 300 ± 20 μm. The top coat was formed on the bond coats using powdered zirconia (ZrO_2_) containing 8 wt % of yttria (METCO 204 C-NS, hereinafter C-NS; Sulzer Metco Holding AG, particle size of 45–125 μm) by the APS process. The thickness of the top coat was approximately *d* = 600 ± 50 μm. The fabrication parameters for the bond and top coats were recommended by the manufacturer (Chrome-Alloying Co. Ltd., Hatfield, UK).

### 2.2. Thermal Fatigue and Thermal-Shock Tests

**Figure 1:**
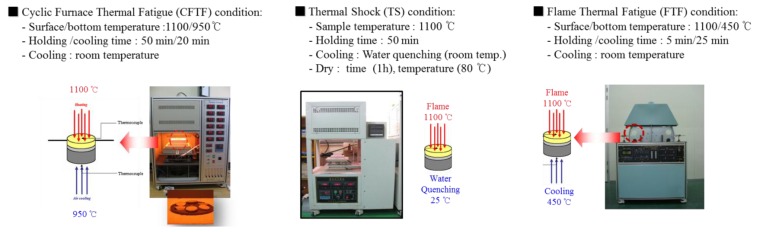
Photos of each test apparatus: Cyclic furnacec thermal fatigue (CFTF), thermal shock test (TS), and flame thermal fatigue (FTF).

A bottom-loading programmable cyclic furnace was used to determine the life cycle of the TBC systems. The cyclic furnace thermal fatigue (CFTF) tests were performed till 1429 cycles in the specially designed furnace: one side of specimen was exposed and the other side air-cooled. The surface temperature of specimen was about 1100 °C with a temperature difference of 150 °C between the top surface and bottom of specimen with a dwell time of 60 min, followed by natural air cooling for 10 min at room temperature. The flame thermal fatigue (FTF) tests using Liquefied Petroleum Gas were also performed till 1429 cycles at a surface temperature of 1100 °C for a dwell time of 5 min, and then the specimen was cooled to room temperature for 25 min. In the FTF tests, the top surface temperature of the specimen was 1100 °C while the bottom surface was 350–500 °C. The failure criterion was defined as 25% buckling or spallation of the top coat in both tests. The TBC specimens were removed at different fractions of their life for cross-sectional studies, while others were observed for signs of failure and were cycled until the failure criterion was met. The TBC specimens for the thermal-shock (TS) tests were annealed using a muffle furnace. After reaching at 1100 °C, the specimens were placed in the furnace. In the TS tests, the specimens were held for 60 min in the furnace and then directly quenched in water for 5 min. Throughout the TS tests, the temperature of the water was between 20 and 35 °C. More than 50% of the region spalled in the top coat was adopted as the criterion for the failure in water-quenched specimens. The TS tests were reported in previous studies while investigating the thermal durability of TBC system [18,19,20,21]. At least five specimens were tested for each condition. The photos of each apparatus for the thermal fatigue and thermal-shock tests are shown in Figure 1.

### 2.3. Characterization

The selected specimens before and after cyclic thermal exposure were preprocessed to observe the cross-sectional microstructure and mechanical properties. The mounted specimens were given a final polish with 1 μm diamond paste. The cross-sectional microstructures of the TBC specimens were observed using a scanning electron microscope (SEM; Model JSM–5610, JEOL, Japan). The thickness of the thermally grown oxide (TGO) layer formed at the interface between the bond and top coats after thermal exposure was measured using the SEM. The phase analysis was performed using an X-ray diffractometer (Model PW 3040, Philips X-pert MPD, Eindhoven, Netherlands). The hardness values of the bond and top coats before and after the thermal exposure were determined using a microindenter (HM-114, Mitutoyo Corp., Kawasaki, Japan) with a Vickers tip for a load of 3 N [22]. To obtain more reliable values, 10 points were tested for each result. The size of the hardness impression was measured by the SEM and all experiments were performed at room temperature. The adhesive strength of each TBC with different bond coats before and after the FTF was measured according to the ASTM standard (ASTM-C-633-01) [23]. The specimen for the adhesive strength was prepared by bonding that to the jig fixture with an epoxy adhesive in the oven at 200 °C for 3 h.

## 3. Results and Discussion

### 3.1. Microstructure of As-Prepared TBCs

**Figure 2:**
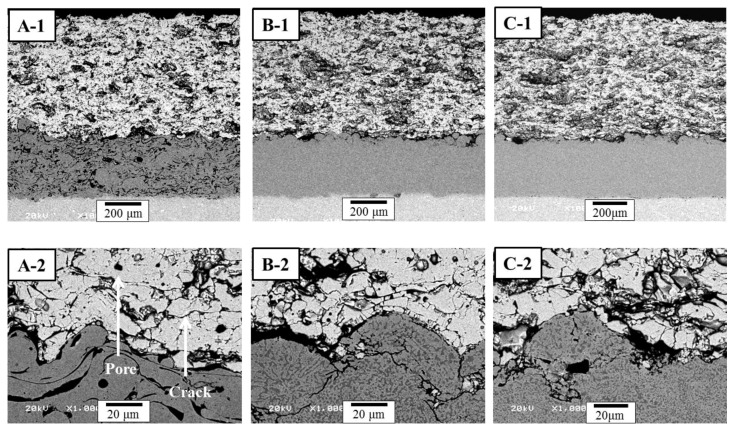
Cross-sectional microstructures of as-prepared thermal barrier coating (TBCs): (**A**) TBC with air-plasma spray (APS) bond coat; (**B**) Thermal barrier coating (TBC) with HVOF bond coat; and (**C**) TBC with low-pressure plasma spray (LPPS) bond coat. Each number indicates the overall and interface microstructures, respectively.

The cross-sectional microstructures of as-prepared TBC specimens are shown in Figure 2, indicating that Figure 2(A-1)–(C-1) are the microstructures of the TBCs with different bond coats prepared by the APS, HVOF and LPPS processes, respectively. The top coats prepared by the APS process showed intrinsic defects, such as pores, unmelted particles, and splat boundaries. In the APS process, many small particles are accelerated by the high-power plasma to impinge on the bond coat to form the top coat. The interface microstructures between the top and bond coats of TBCs formed in this study are shown in Figure 2. The interface of the TBCs (Figure 2(A-2)–(C-2)) showed irregular shapes at the interface between the top and bond coats, without the formation of a TGO layer and cracking between the top and bond coats. The bond coats prepared by the HVOF and LPPS exhibited similar microstructure with a dense microstructure and no oxide formation, whereas the APS bond coat featured high levels of visible oxides. 

It is well known that the microstructure and roughness of the bond coat are significantly affected by both the composition as well as powder size. In addition, the interface roughness (surface roughness) of the bond coat in turn is one of the most important factors that affect the lifetime of TBC systems [24,25]. However, in other study, it was reported that the APS TBC lifetime was independent of average surface roughness [26]. In this study, the feedstock powders for each bond coat were selected to get an optimal microstructure in each coating process, which were recommended by the manufacturer (Chrome-Alloying Co. Ltd., Hatfield, UK). Even though each TBC system showed a little different interface microstructure in a microscopic viewpoint, the interface structure was much similar with each other in a macroscopic viewpoint. Oxides in the APS bond coat are generally seen as dark, elongated phases that appear as strings in the microstructure, parallel to the substrate. Oxides are produced by particle/atmosphere interaction and/or heating of the coating surface during deposition. Interaction of the hot particles with their surrounding environment, usually air, leads to oxide film on particle surfaces. Longer dwell times and higher particle temperatures increase the thickness of the oxide layer on the particles, producing higher concentrations of oxide stringers within the bond coat [27].

### 3.2. Lifetime of TBC Systems 

**Figure 3:**
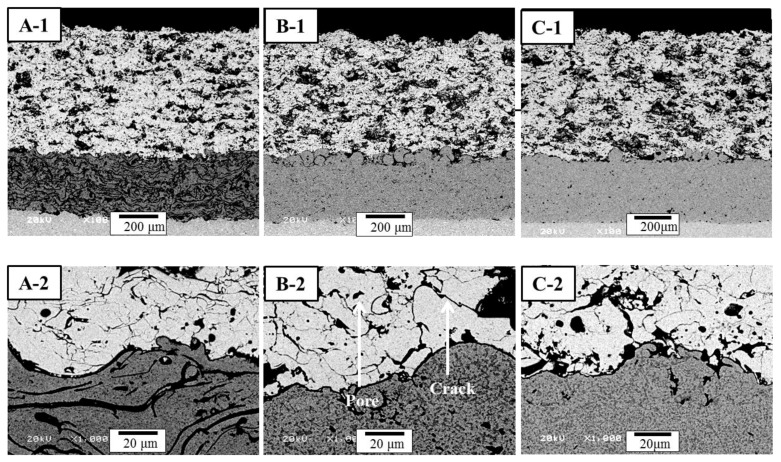
Cross-sectional microstructures of TBCs after FTF tests for 1429 cycles: (**A**) TBC with APS bond coat; (**B**) TBC with HVOF bond coat; and (**C**) TBC with LPPS bond coat. Each number indicates the overall and interface microstructures, respectively.

Cross-sectional microstructures of different bond coats deposited by the APS, HVOF, and LPPS are shown in Figure 3 after the FTF tests for 1429 cycles. After the thermal fatigue tests for 1429 cycles using the FTF apparatus, the interface microstructure of each TBC showed a sound condition without cracking or delamination. The TGO layer was not fully developed after 1429 cycles, because of the relatively short thermal exposure time (5 min) and the lower temperature on the bond coat (the temperature of bottom surface: 350–500 °C), showing a thickness for the TGO layer in the range of 2–3 μm. The interface microstructures were densified owing to resintering during the thermal exposure to 1429 cycles, even though the thermal exposure time was just 119 h. The interface microstructures of each TBC system after the FTF were much similar with each other, compared with the as-prepared TBC. 

**Figure 4:**
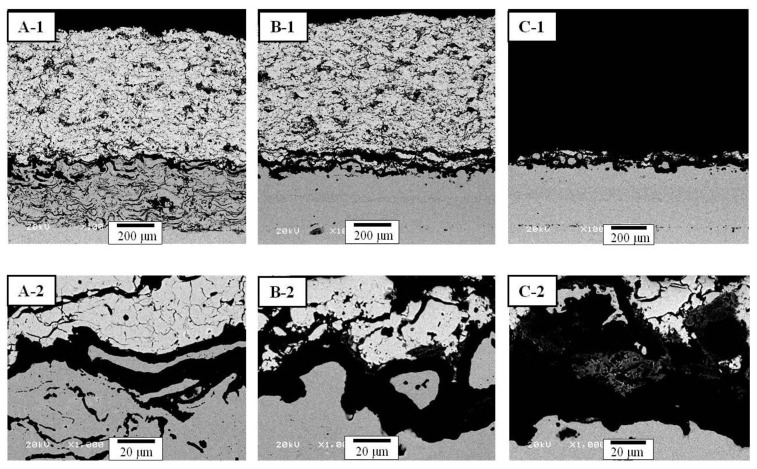
Cross-sectional microstructures of TBCs after CFTF tests: (**A**) TBC with APS bond coat; (**B**) TBC with HVOF bond coat; and (**C**) TBC with LPPS bond coat. Each number indicates the overall and interface microstructures, respectively.

**Figure 5:**
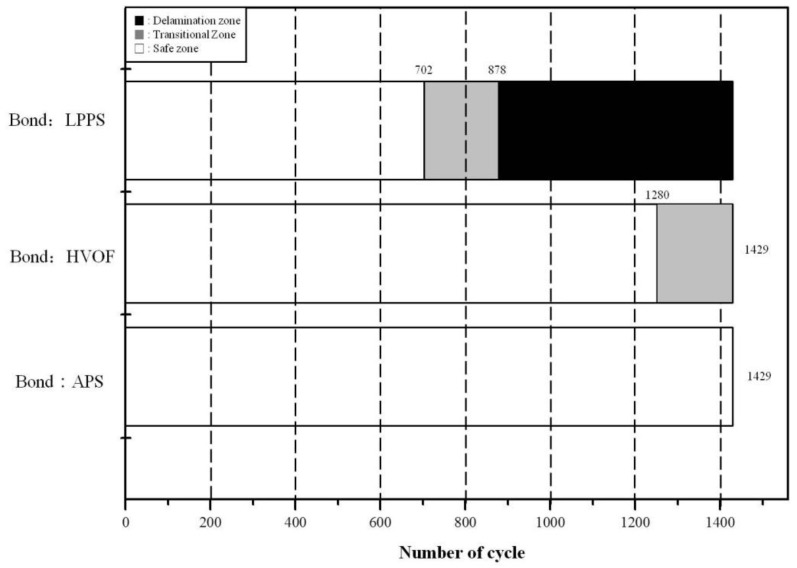
The lifetime of TBCs with different bond coats after CFTF tests as a function of cycle number.

The cross-sectional microstructures of different bond coats deposited by the APS, HVOF, and LPPS processes are shown in Figure 4 after the CTFT tests. The TBC with the APS bond coat (Figure 4(A-1)) showed defects, such as the horizontal and vertical cracks, in the top coat, TGO layer at the interface, and oxidation in the bond coat after the CTFF for 1429 cycles. In the case of TBC with the HVOF bond coat (Figure 4(B-1)), a thick and long crack near the interface between the top coat and TGO layer was newly developed. The TBC with the LPPS bond coat (Figure 4(C-1)) was delaminated after 780 cycles, but no delamination was observed in other TBCs. The nominal thickness of the TGO layer in the TBC with the APS bond coat (Figure 4(B-1)) was 10.5 mm, whereas the nominal thickness of the TGO layer was 16.5 mm for the TBC with the HVOF bond coat (Figure 4(B-2)). The nominal thickness for the TGO layer for the TBC with the LPPS bond coat was 13.3 mm (Figure 4(C-2)), after delaminating at 780 cycles. The oxidation of the bond coat leads to a change in sign of stresses due to the smaller coefficient of thermal expansion (CTE) of the TGO layer. It is assumed that small cracks formed in the regions of initial tensile stress at the peak tips before and grow into the valleys after stress conversion [28].The lifetime of different bond coats deposited by the APS, HVOF, and LPPS processes is shown in Figure 5 after the CTFT tests. The result indicates that the structural effectiveness is one of important factors for thermal durability of TBC system. 

**Figure 6:**
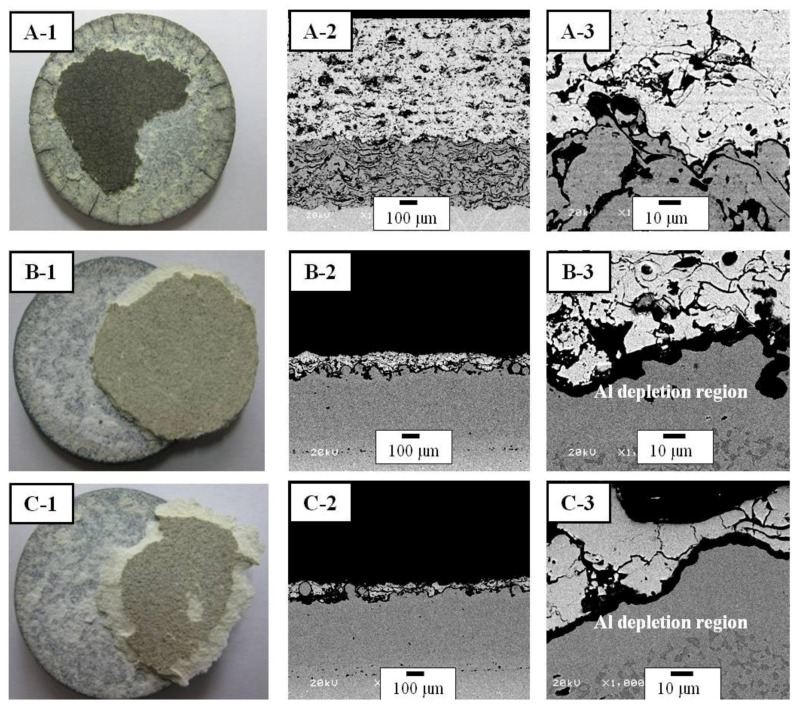
Surface micrographs and cross-sectional microstructures of TBCs after TS tests: (**A**) TBC with APS bond coat; (**B**) TBC with HVOF bond coat; and (**C**) TBC with LPPS bond coat. Each number indicates surface micrographs, cross-sectional interface microstructures between the top and bond coats, and high magnification interface microstructures, respectively.

The surface micrographs and cross-sectional microstructures of TBCs after the TS tests are shown in Figure 6. The surface micrographs showed a delamination of spalling mode at the interface between the bond and top coats. This may be due to the large temperature difference between the substrate and the top coat surface during the quenching and annealing processes. The temperature difference causes thermal stresses at the interface of the bond and top coats. The mismatch in the CTEs between the top and bond coats or oxidation of the bond coat leads to delamination and failure. The TBCs with the APS bond coat were delaminated in the range of 145–159 cycles, whereas the TBCs with the HVOF bond coat were delaminated after 30–36 cycles. In addition, the TBCs with the LPPS bond coat were delaminated after 42–46 cycles. In the TBCs with the APS bond coat, the radial cracks were created from the edge of specimens due to the relatively higher number of cycles. The TBCs with the bond coats by the HVOF and LPPS showed the Al depletion region between the bond and top coats. Usually the depletion of Al in the bond coat after oxidation was about 6 wt % for the VPS bond coat and about 4 wt % for the APS bond coat at TBC failure, indicating that the lifetime of TBCs with the APS bond coat was superior to that of TBCs with the VPS bond coat [26]. The result of previous study is in agreement with our results of the CFTF and TF. 

**Figure 7:**
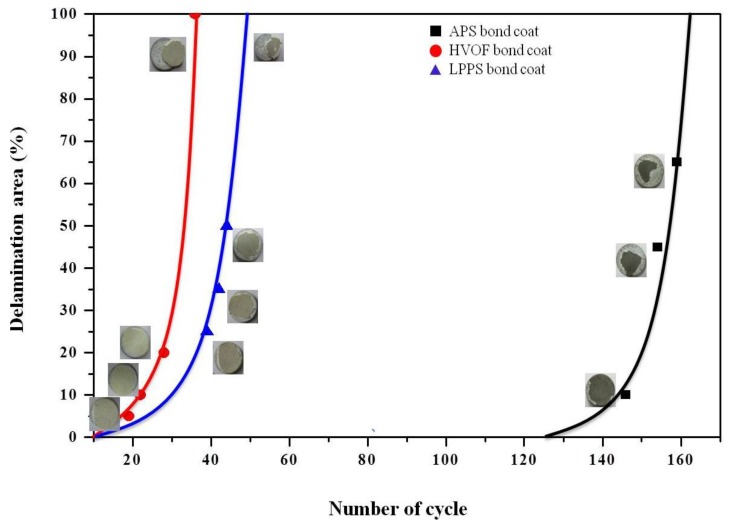
Variation of delamination area with thermal fatigue cycle in thermal-shock tests. The solid curves are empirical data fits.

Evolution of the delamination area during the TS tests is shown in Figure 7 as a function of cycle number. The TBC with the APS bond coat started to be delaminated after 130 cycles, where the delamination area was only 5%. After 155 cycles, the TBC was delaminated more than 60%. However, the TBC with the APS bond coat was not completely delaminated. The TBC with the HVOF bond coat started to be delaminated after 18 cycles, where the area of delamination was 10%. After 36 cycles, the top coat was completely delaminated. In the case of the TBC with the LPPS bond coat, the delamination started to occur after 40 thermal cycles, which was completed after 52 cycles. In all cases, the top coats were delaminated more that 50% in the range of 10–25 cycles after starting to delaminate, meaning that control of the initial delamination is more important in enhancing the lifetime performance of TBCs. The lifetime of TBCs with different bond coats after the thermal fatigue and thermal-shock tests are shown in Table 1.

**Table 1:** The lifetime of thermal barrier coating (TBCs) with different bond coats after the thermal fatigue and thermal-shock tests.

Test species	ASP bond coat	HVOF bond coat	LPPS bond coat
Cyclic furnace thermal fatigue (CFTF)	1429 cycles	1429 cycles	1429 cycles
Flame thermal fatigue (FTF)	1429 cycles	1000~1429 cycles	780 cycles
Thermal shock test (TS)	159 cycles	36 cycles	46 cycles

### 3.3. Phase Analysis and TGO after Thermal Exposure

**Figure 8:**
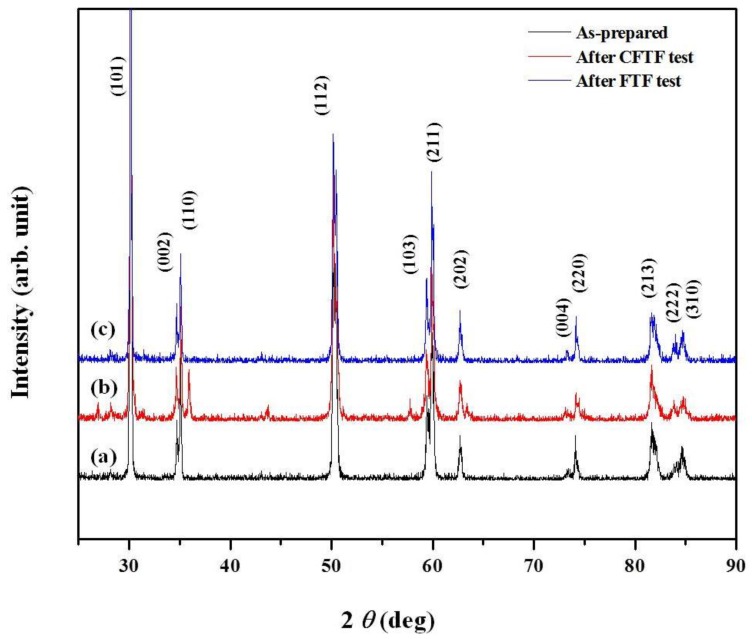
X-ray diffraction (XRD) pattern of TBCs of as-prepared and after cyclic thermal exposure.

X-ray diffraction (XRD) patterns were investigated to confirm the phase identification of the TBCs before and after the tests as shown in Figure 8. All of the peaks were tetragonal and cubic phases without any monoclinic phase before and after thermal exposure. Any phase transformation causing cracks and defects in the TBC was not observed after all tests performed in this study. It is important to suppress the monoclinic phase to enhance the thermal stability. It was verified that the TBCs prepared in this work was not affected by any phase transformation.

**Figure 9:**
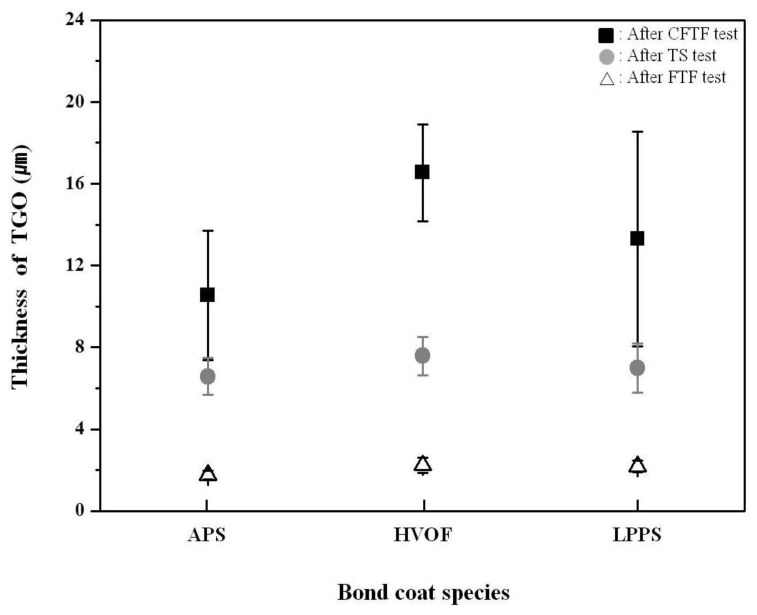
Thickness of thermally grown oxide (TGO) layer with bond coat species after different thermal exposure tests. Black, gray, and white marks indicate the TGO thickness values after the CFTF, TS, and FTF tests, respectively.

The thickness of the TGO layer for the three TBC systems after each test are shown in Figure 9. After the FTF tests, the TGO layer was not fully developed after 1429 cycles, owing to the relatively short thermal exposure time (119 h), showing nominal thicknesses of the APS, HVOF, and LPPS specimens as 1.8, 2.2, and 2.3 μm, respectively. The TBCs with the bond coats prepared by the APS and HVOF processes showed nominal thicknesses of the TGO layer as 10.5 and 16.5 μm, respectively, after the CFTF tests for 1429 cycles. In the case of the TBC with the LPPS bond coat, the nominal thickness of the TGO layer was 13.3 μm after the CTFT tests at TBC delamination (780 cycles). The difference in the thickness of the TGO layer between the FTF and CFTF tests was due to different thermal exposure times and the temperature of bottom surface. If the exposure time is increased at higher temperature, the thickness of the TGO layer would be increased in a certain cycles. Also, the temperature of bottom surface affects the growth of the TGO layer, resulting in a thicker thickness of TGO layer at higher temperature. After the TS tests, the thickness of the TGO layer did not show a difference with bond coat species in the FTF tests, showing nominal thicknesses of 6.6, 7.6, and 7.0 μm, for the APS, HVOF, and LPPS, respectively.

### 3.4. Mechanical Properties

**Figure 10:**
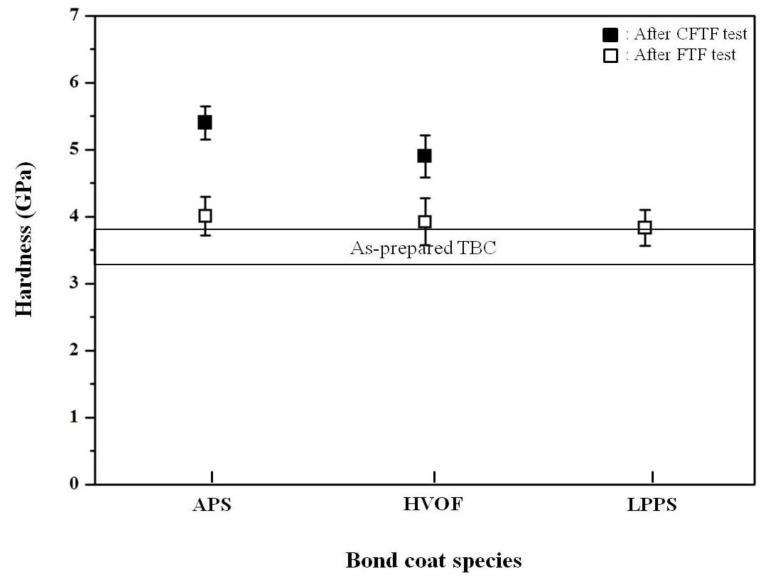
Hardness values of top coats before and after cyclic thermal exposure. Indentation for hardness was conducted on the sectional plane with a load of 3 N. The nominal value of as-prepared TBC was indicated inside figure. Filled and open marks indicate the hardness values after the CFTF and ETF tests, respectively.

**Figure 11:**
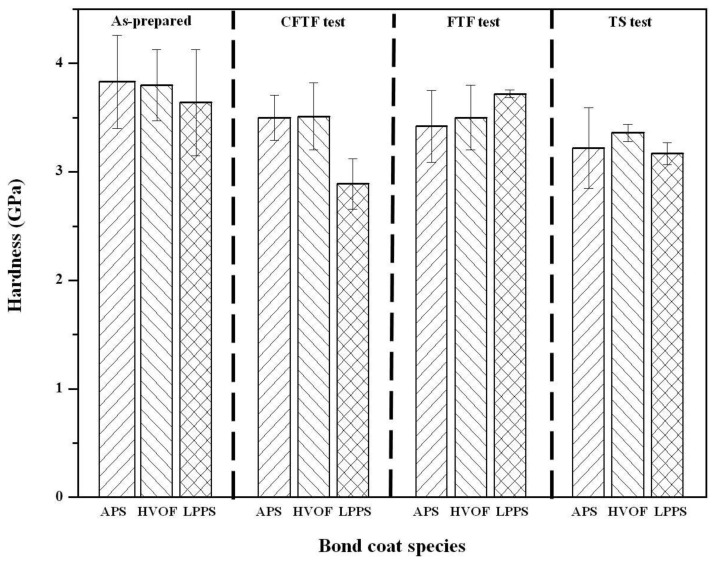
Hardness values of bond coats before and after cyclic thermal exposure. Indentation for hardness was conducted on the sectional plane at 3 N.

The hardness values of the top coats before and after thermal exposure were measured using a Vickers indentation method (Figure 10). The indentation tests were conducted on the sectional plane with a load of 3 N at room temperature. The hardness values of the top coats in the as-prepared TBCs were determined to be 3.45 ± 0.23 GPa (mean ± standard deviation). After the FTF tests for 1429 cycles, the hardness values of the top coats were increased to 4.01 ± 0.29, 3.92 ± 0.35, and 3.83 ± 0.27 GPa for the bond coats prepared by the APS, HVOF, and LPPS processes, respectively. The increase in the hardness values after thermal exposure was due to the reduction of pores and defects [29,30], which is in good agreement with the microstructural evolution in Figure 2, Figure 3 and Figure 4. The microstructural evolution of the top coat prepared by the APS was more advanced, resulting in the disappearance of the pores and splat boundaries. The TBCs with the bond coats prepared by the APS and HVOF processes showed hardness values of 5.40 ± 0.25 and 4.90 ± 0.32 GPa, respectively, after the CTFT tests for 1429 cycles. The TBC with the LPPS bond coat was delaminated after the ETF tests for 780 cycles and hence there was no hardness value. After the TS tests, all of the TBCs were delaminated at the interface between the top and bond coat, therefore there was no hardness value in the top coat. The hardness values of the bond coats before and after the thermal exposure tests are shown in Figure 11. In the bond coats, the hardness values were modestly decreased, independent of bond coat species, compared with those of as-prepared bond coats. This result agrees with that of previous investigation [29,30]

**Figure 12:**
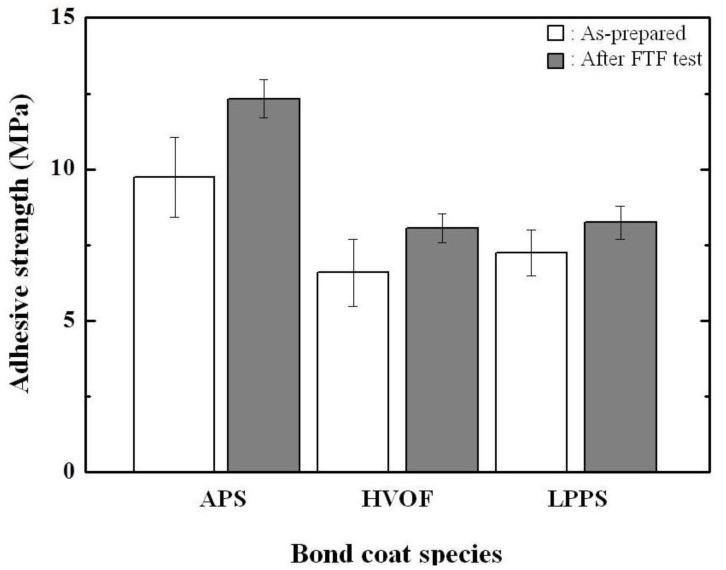
Adhesive strength values of TBCs before and after the FTF.

The origin of delamination at the interface between the bond and top coats is strongly related to the adhesive strength. Therefore, the adhesive strength values were measured for the TBCs before and after the FTF tests, which are shown in Figure 12. The adhesive strength values of the as-prepared TBCs with the bond coats prepared by the APS, HVOF, and LPPS processes were determined to be 9.73 ± 1.33 (mean ± standard deviation), 6.58 ± 1.11, and 7.23 ± 0.76 MPa, respectively. All of the TBCs were completely delaminated near the interface between the top and bond coats. The adhesive strength values of the top coats are increased to 12.32 ± 0.63, 8.58 ± 1.12, and 8.24 ± 0.54 MPa for the TBCs with the bond coats prepared by the APS, HVOF, and LPPS processes, respectively, after the FTF tests for 1429 cycles. The results indicate the interface stability of the TBCs with the APS bond coat is better than the TBCs with the HVOF or LPPS bond coat. Therefore, the TBCs with the APS bond coat provided superior TBC lifetime in cyclic thermal exposure.

## 4. Conclusions 

The influences of bond coat species on the thermal fatigue behavior, especially on the thermal durability, of the top coat prepared by the APS were investigated through cyclic thermal exposure. The bond coats were successfully formed by the APS, HVOF, and LPPS processes. In the FTF tests, the interface microstructure of each TBC showed a sound condition without cracking or delamination, independent of bond coat species. The TGO layer was not fully developed, owing to the relatively short thermal exposure time. The CFTF tests showed no delamination for the TBCs with the APS bond coat and partial delamination for the TBCs with the HVOF bond coat, whereas the TBC with the LPPS bond coat was delaminated after 780 cycles. The nominal thicknesses of TGO layers were in the range of 10.5–16.5 μm and 6–8 μm for the CFTF and FTF, respectively. The thickness of TGO layer was strongly affected by the exposure time and the temperature difference between the surface and bottom, depending on the test apparatus or test method. The TS tests showed that the TBCs with bond coats prepared by the APS, HVOF, and LPPS processes were fully delaminated after 159, 36, and 42 cycles, respectively, indicating that the TBC system with the APS bond coat is better thermal stability. In the XRD patterns before and after thermal exposure, all of the peaks were the tetragonal and cubic phases without any monoclinic phase. The hardness values of the top coats were increased to 4.01 ± 0.29, 3.92 ± 0.35, and 3.83 ± 0.27 GPa for the TBCs with the bond coats prepared by the APS, HVOF, and LPPS processes, respectively, after the FTF tests for 1429 cycles. The hardness values of the bond coats were slightly decreased after thermal exposure, independent of bond coat species. The adhesive strength values of the as-prepared TBCs were increased after the FTF tests, showing the highest values for the TBC with the APS bond coat. In the TBC system with the top coat prepared by the APS, the APS bond coat was more efficient in improving thermal durability than those with the bond coats prepared by the HVOF and LPPS in the cyclic thermal exposure environments. 
